# Case report: Molecular profiling facilitates the diagnosis of a challenging case of lung cancer with choriocarcinoma features

**DOI:** 10.3389/fonc.2024.1324057

**Published:** 2024-03-25

**Authors:** Hui Li, Xin Hu, Matthew S. Ning, Gregory N. Fuller, John M. Stewart, Jared C. Gilliam, Jia Wu, Xiuning Le, Ara A. Vaporciyan, J. Jack Lee, Don L. Gibbons, John V. Heymach, Andrew Futreal, Jianjun Zhang

**Affiliations:** ^1^ Department of Thoracic/Head and Neck Medical Oncology, The University of Texas MD Anderson Cancer Center, Houston, TX, United States; ^2^ Department of Imaging Physics, The University of Texas MD Anderson Cancer Center, Houston, TX, United States; ^3^ Department of Genomic Medicine, The University of Texas MD Anderson Cancer Center, Houston, TX, United States; ^4^ Department of Thoracic Radiation Oncology, The University of Texas MD Anderson Cancer Center, Houston, TX, United States; ^5^ Department of Pathology, The University of Texas MD Anderson Cancer Center, Houston, TX, United States; ^6^ Caris Life Sciences, Phoenix, AZ, United States; ^7^ Department of Thoracic and Cardiovascular Surgery, The University of Texas MD Anderson Cancer Center, Houston, TX, United States; ^8^ Department of Biostatistics, The University of Texas MD Anderson Cancer Center, Houston, TX, United States; ^9^ Department of Molecular and Cellular Oncology, The University of Texas MD Anderson Cancer Center, Houston, TX, United States

**Keywords:** lung cancer with choriocarcinoma features, whole transcriptome sequencing, whole exome sequencing, immune checkpoint inhibitors, nivolumab, ipilimumab

## Abstract

Accurate diagnoses are crucial in determining the most effective treatment across different cancers. In challenging cases, morphology-based traditional pathology methods have important limitations, while molecular profiling can provide valuable information to guide clinical decisions. We present a 35-year female with lung cancer with choriocarcinoma features. Her disease involved the right lower lung, brain, and thoracic lymph nodes. The pathology from brain metastasis was reported as “metastatic choriocarcinoma” (a germ cell tumor) by local pathologists. She initiated carboplatin and etoposide, a regimen for choriocarcinoma. Subsequently, her case was assessed by pathologists from an academic cancer center, who gave the diagnosis of “adenocarcinoma with aberrant expression of β-hCG” and finally pathologists at our hospital, who gave the diagnosis of “poorly differentiated carcinoma with choriocarcinoma features”. Genomic profiling detected a KRAS G13R mutation and transcriptomics profiling was suggestive of lung origin. The patient was treated with carboplatin/paclitaxel/ipilimumab/nivolumab followed by consolidation radiation therapy. She had no evidence of progression to date, 16 months after the initial presentation. The molecular profiling could facilitate diagnosing of challenging cancer cases. In addition, chemoimmunotherapy and local consolidation radiation therapy may provide promising therapeutic options for patients with lung cancer exhibiting choriocarcinoma features.

## Introduction

Lung cancer remains as the leading cause of cancer-related deaths worldwide. Non-small cell lung cancer (NSCLC) accounts for almost 85% of all lung cancers, with adenocarcinoma and squamous cell carcinoma as the most common histologic subtypes of NSCLCs (1). NSCLCs can present as other rare histologies (2). Accurate histopathological diagnosis is crucial, as treatment and prognosis vary among different subtypes (3). Currently, the histopathological diagnosis is primarily based on morphological characteristics and immunohistochemical patterns. Although it remains as the gold standard in cancer diagnosis, it has important limitations, particularly for rare lung cancer subtypes. This report highlights the role of molecular profiling in differential diagnosis of a rare lung cancer subtype.

## Case presentation

A 35-year-old white female, never smoker, presented to the emergency room (ER) with complaints of a syncope episode and right arm pain in September 2022. She had been experiencing right-sided neuropathic symptoms for 2 months prior to ER visit. Computed tomography (CT) scan of the head revealed a left frontal hypodensity, and brain magnetic resonance imaging (MRI) showed a 2.0 x 1.6 x 1.6 cm peripherally enhancing lesion in the peripheral aspect of the left postcentral gyrus, which was suspected to be an intracranial abscess. Subsequently, the patient underwent frontal craniotomy. However, the pathology report revealed a pleomorphic epithelial tumor with extensive necrosis and scattered multinucleated cells. Immunohistochemical staining was positive for pancytokeratin, CK7, β-hCG and focal cytoplasmic staining for inhibin. The cells were negative for GATA3, CK20, TTF-1, and PAX 8. The Ki-67 was approximately 60%. These results were consistent with metastatic choriocarcinoma.

**Figure 1:**
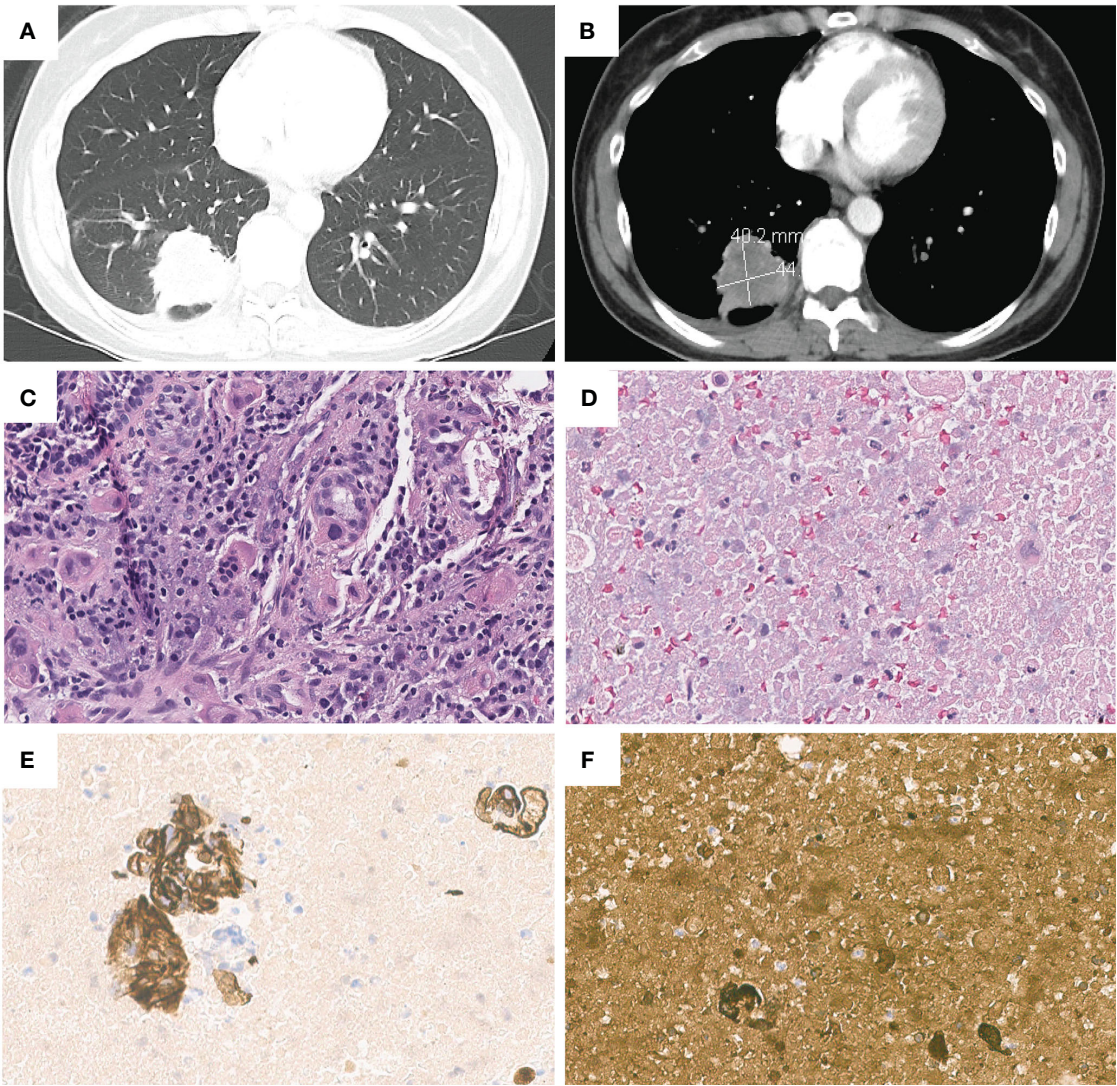
Chest contrast-enhanced computed tomography images revealed a 4.0*4.4 cm right lower lobe lung mass, in **(A)** lung window and **(B)** mediastinal window. Pathologic and immunohistochemical findings in lung cancer with choriocarcinoma features. **(C)** H&E staining of lung tumor. **(D)** H&E staining of metastatic lymph node(11R). Lymph node(11R) showed positive staining for **(E)** CK7 and **(F)** β-hCG.

The patient’s laboratory results were mostly unremarkable, with normal alpha-fetoprotein (AFP) levels. There was a slight elevation in serum β-hCG levels from 171 to 248 mIU/mL (Reference range 0-5 mIU/mL) during the first week following the frontal craniotomy. Further examinations, including pelvic and transvaginal ultrasound, abdominal and pelvic CT did not identify any masses. However, CT scan of the chest revealed a 4.0 x 4.4 cm opacity in the right lower lobe (**Figures 1A, B**), leading to an EBUS for the biopsy of the lung mass and mediastinal nodes. The results showed poorly differentiated carcinoma, compatible with choriocarcinoma. CK7 and β-hCG staining were positive and the tumor had morphologic features like the brain lesion (**Figures 1C–F**). PET/CT scan showed a hypermetabolic mass lesion in the right lower lobe along with hypermetabolic lymph nodes in the neck, mediastinum, bilateral hilar, and right axillary regions.

The patient was initially treated as metastatic choriocarcinoma and received one cycle of etoposide and cisplatin chemotherapy (EP) from her local oncologist. She proceeded to seek a second opinion at a university hospital, where the pathologists re-evaluated the tissues from the brain metastasis, right lower lobe mass, and subcarinal lymph node. Immunostaining was negative for CDX2, Glypican, TTF-1, P63, and Napsin, while positive for β-hCG, claudin-4 and Ber-Ep4. PD-L1 staining could not be completed due to insufficient tissue samples. The final diagnosis was “adenocarcinoma with aberrant expression of β-hCG” and the primary site of the tumor was undetermined. Finally, genomic profiling of brain metastasis revealed KRAS p.G13R and GNAS p.R201H mutations among others (**Table 1**). The same KRAS and GNAS mutations were also detected by liquid biopsy using Guardant 360 platform (**Table 1**).

**Table 1:** Genetic profile results in lung cancer with choriocarcinoma features.

Biopsy Samples	Biomarkers	Details	Variant Allele Fraction
Brain metastasis	KRAS	p.G13R	17.8%
	GNAS	p.R201H	8.2%
	ERBB2(Her2)	p.R1053G	45.8%
	ANXA7	p.R479*	41.9%
	BAP1	p.V439M	7.2%
	TIGIT	p.V100M	46.7%
	WNK1	p.C733fs	10.5%
	ZRSR2	p.S447_R448insQS	39.0%
	Tumor Mutational Burden	3.7 mutations/Mb	–
	Microsatellite instability	Stable	–
Blood	KRAS	p.G13R	0.3%
	GNAS	p.R201H	0.2%
Lung tumor	KRAS	p.G13R	20.0%
	GNAS	p.R201H	9.0%
	ERBB2(Her2)	p.R1053G	50.0%
	BAP1	p.V439M	11.0%
	DDB2	p.R20K	44.0%
	DOT1L	p.S1061C	50.0%
	NSD3	p.R326W	48.0%
	POLQ	p.K58Q	48.0%
	THRAP3	p.H761R	48.0%
	TNFAIP3	p.R761H	40.0%
	ZRSR2	p.S447_R448insQS	61.0%
	Tumor Mutational Burden	Low, 1.0 mutations/Mb	–
	Microsatellite instability	Stable	–
	Genomic loss of Heterozygosity (LOH)	Low, 2.0% of tested genomic segments exhibited LOH	–

The patient sought to a third opinion at our hospital. Our gynecologic oncologists reviewed her obstetric and gynecologic history and discovered that she had successfully delivered three children, each at full term without complications. Her most recent delivery was 4.5 years ago, and she had consistently experienced regular monthly periods without intermenstrual spotting. The peak serum β-hCG levels reached 287 mIU/mL (Reference range 0-5 mIU/mL) and declined to negative shortly after treatment. This was significantly lower than the typical β-hCG levels associated with choriocarcinoma, which can reach up to 100,000 mIU/mL. In addition, there was no indication of uterine or ovarian abnormalities on CT imaging. Thus, our gynecologic oncologists determined that the primary malignancy is unlikely to be of gynecologic origin. Our thoracic pathologists conducted a thorough review of the patient’s slides, reporting it as poorly differentiated carcinoma with features of choriocarcinoma. However, there were different opinions among other pathologists, who were inclined to consider it as adenocarcinoma with β-hCG production.

Following the first cycle of etoposide and cisplatin, the primary tumor showed a slight increase in size, growing from 4.4cm to 5.0cm. Based on the patient’s clinical, radiologic and molecular profiling results, the patient was transitioned to the CheckMate 9LA regimen (carboplatin/paclitaxel/ipilimumab/nivolumab) (4). The tumor size decreased 21% after two cycles of treatment. The serum β-hCG level declined to normal. Given her young age and relatively limited disease burden, the patient was referred to the radiation oncology team to consider local consolidation based on previous studies (5, 6). The patient underwent EBUS prior to radiation and fine needle aspiration, brushing tissue, and transbronchial biopsy of multiple thoracic lymph nodes from stations 11L, 4L, 2L, 2R, 4R, 11RS and 11RI came back negative for malignant cells. She subsequently underwent volumetric modulated arc therapy (VMAT) to the right lower lung and mediastinum in January 2023. The patient returned to her local oncologist. She discontinued chemotherapy and received ipilimumab and nivolumab until April 2023, and then continued with nivolumab monotherapy. Her most recent brain MRI and chest scan in January 2024 revealed good disease control without evidence of recurrence.

**Figure 2:**
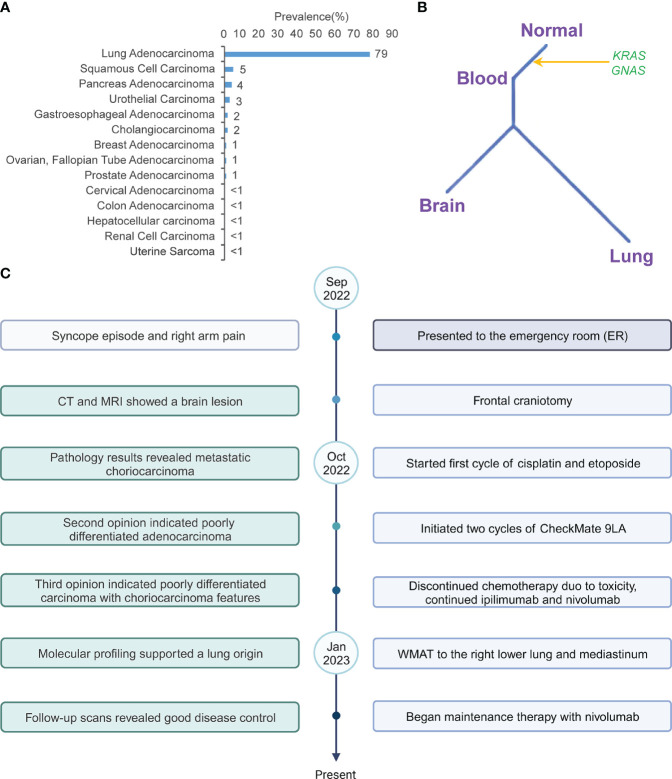
**(A)** The prevalence of cancer types based on the tumor’s molecular signature, including all the mutations (Data from Caris). **(B)** Phylogenetic tree of lung cancer with choriocarcinoma features. **(C)** Timeline of the patient’s diagnosis and treatments.

Of note, molecular profiling using whole transcriptome sequencing (WTS) and whole exome sequencing (WES) by Caris was also consistent with lung cancer diagnosis. The same KRAS G13R and GNAS R201H among others were identified in lung tumor (**Table 1**). Additionally, a cancer-type similarity assessment that compared patient’s tumor against the signatures across 21 distinct common cancer types in the Caris database revealed that the most likely cancer type was lung adenocarcinoma (**Figure 2A**).

## Discussion

Choriocarcinoma is an aggressive tumor, which is classified into gestational and non-gestational choriocarcinoma. Gestational choriocarcinoma originates from trophoblastic cells and usually associates with pregnancy events including hydatidiform moles and production of β-hCG (7). Non-gestational choriocarcinoma typically is a mixed germ cell tumor, showing differentiation towards trophoblastic structures. Non-gestational choriocarcinoma has been reported in extragonadal sites. These tumors typically occur in midline locations such as the mediastinum, retroperitoneum, or brain (8). Interestingly, trophoblastic differentiation has been observed in tumors originating from various organs. This has led to a debate regarding whether these cancers should be classified as extragonadal choriocarcinoma (9, 10). These types of carcinomas have been reported in different organs, including liver, stomach, cervix, lung and others (11–14).

Lung cancer exhibiting choriocarcinoma features or trophoblastic differentiation is a rare occurrence, and it has been reported in some case reports (**Supplementary Table 1**). However, it’s evident that there is substantial controversy among pathologists regarding the terminology used for this rare subtype of lung cancer, commonly referred to as “primary pulmonary choriocarcinoma (PPC)”. Alternative terms such as “primary choriocarcinoma of the lung”, “lung tumors with trophoblastic morphology” and “lung adenocarcinoma with choriocarcinomatous features” were also employed in published literature. In our case, the biopsies from brain metastasis were reported by local pathologists as “metastatic choriocarcinoma”. Subsequent biopsies of the lung and lymph nodes were reported by local pathologists as “suggestive of choriocarcinoma”. Pathologic consultations at two academic cancer centers reported it as adenocarcinoma with aberrant expression of β-hCG versus poorly differentiated carcinoma with choriocarcinoma features or adenocarcinoma with β-hCG production.

Diagnosis of this rare subtype of lung cancer is challenging, especially in female patients. In addition to particular morphological feature and detection of β-hCG in cancer cells, it is imperative to rule out any prior gynecologic cancers and molar pregnancy in the patient’s medical history (15). It’s noteworthy that typical immunohistochemical markers found in lung adenocarcinoma, such as TTF-1 and Napsin A, were negative in lung cancer with choriocarcinoma features. TTF-1, that plays a crucial role in maintaining terminal respiratory unit cell function in the lung, and Napsin A, an aspartic proteinase involved in surfactant protein maturation (16) may provide valuable information facilitating the diagnosis of primary lung adenocarcinoma. However, they are not always positive in cancers of lung origins. Indeed, both markers were negative in our case, which was consistent with other reported cases (15, 17–19). Taken together, these highlight the profound challenges in identifying the tumor’s origin of rare histologic subtypes.

Clinically, the absence of abnormal bleeding, uterine or ovarian abnormalities and low serum β-hCG levels in our case was against the initial diagnosis of metastatic choriocarcinoma by local pathologists. Although the primary site of the tumor could not be determined based on morphology and IHC staining, the molecular profiling including the cancer gene mutations and transcriptomic features was suggestive of lung origin. The notable mutations detected in this tumor included KRAS G13R and GNAS R201H. KRAS is one of the most common mutated oncogenes and frequently detected in colorectal adenocarcinoma, lung adenocarcinoma, multiple myeloma and pancreatic adenocarcinoma (20, 21). In lung adenocarcinoma, KRAS is the most prevalent cancer driver, with about 35.5% of patients harboring a KRAS mutation (22). On the other hand, KRAS mutations are rarely observed in either gestational or non-gestational choriocarcinomas originating from germ cells (23, 24). KRAS mutations have only been reported in extragonadal choriocarcinomas, such as duodenal choriocarcinoma (25). The tumor in our case carried a pathogenic KRAS G13R mutation, which was supportive of lung origin. Furthermore, the transcriptomic profiling data was also suggestive of lung origin (**Figure 2A**). Taken together, these results suggested that genetic profiling may facilitate the diagnosis and identification of organ origin of rare tumors such as lung cancer with choriocarcinoma features.

Due to its scarcity, the genomic landscape of lung cancer with choriocarcinoma features has not been defined. EGFR L858R, EGFR V774M (17, 18) and TP53 C275G, R273L, V73fs or D281E (17, 26–28) have been reported previously. The case in our study is the first case with KRAS mutation. Another interesting mutation is GNAS p.R201H, an oncogenic activating mutation in alpha-subunit of the stimulatory G protein (Gsα) that caused constitutive Gsα signaling. GNAS mutations have been identified in many epithelial tumors, such as pancreatic cancer and colon cancer. GNAS mutated tumors frequently harbored concurrent mutations in the Ras/Raf pathway, such as KRAS mutations (29). While uncommon, GNAS p.R201H mutation has been detected in lung adenocarcinoma (30). On the other hand, GNAS mutations are also rarely detected in gestational and non-gestational choriocarcinomas. We leveraged the molecular profiling data from lung tumors, brain metastasis and ctDNA to investigate the genomic evolutionary pattern in this case. All tumor samples carried identical KRAS and GNAS mutations, indicating that these mutations were early events in tumor development (**Figure 2B**).

Compared to other NSCLC subtypes, lung cancer with choriocarcinoma features is associated with a very poor prognosis, with a 5-year survival rate of less than 5% (31). This rare type of lung cancer tends to present with widespread metastases and to progress rapidly (15). Due to the rarity, there is no evidence to guide optimal treatment. Chemotherapy including BEP (bleomycin, etoposide and cisplatin) and EMA-CO (etoposide, methotrexate, actinomycin-D, cyclophosphamide, vincristine) (15, 32) is the commonly used therapeutic modality. Surgery or radiation in combination with chemotherapy was also employed for lung cancer with choriocarcinoma features of early stages (33).

Immune checkpoint inhibitors (ICIs) have revolutionized the therapeutic landscape across different cancer types including NSCLC. However, the use of ICIs in gestational choriocarcinoma remains under investigation. Notably, high levels of PD-L1 expression were observed in gestational choriocarcinoma (34), leading to the potential utilization of ICIs such as anti-PD1/PD-L1 as treatment options. Case reports and small sized clinical trials have explored the potential of ICIs, particularly pembrolizumab, for patients who progressed after initial chemotherapy (35, 36). Favorable outcomes, including complete responses, were observed in cases with strong PD-L1 expression (often 90%-100%) (37–43). Additionally, toripalimab and tirelizumab showed promise in chemo-resistant choriocarcinoma (44, 45).In a phase 2 clinical trial, the combination of camrelizumab and apatinib showed potential for chemo-resistant choriocarcinoma, with a 50% complete response rate (46). In the TROPHIMMUN trial, avelumab also demonstrated curative potential in 50% chemotherapy-resistant gestational trophoblastic tumors, including cases of choriocarcinoma (47). Despite these encouraging findings, the variability in response rates and long-term benefits of ICIs in gestational choriocarcinoma require further investigation.

Evidence concerning the using of ICIs in non-gestational choriocarcinoma, especially extragonadal cases, was limited. In a case report involving non-gestational choriocarcinoma, pembrolizumab was utilized as a second-line treatment following the initial EMA-CO regimen. Unfortunately, the patient experienced rapid progression and subsequently returned to chemotherapy. Despite attempting pembrolizumab once more, the treatment did not yield a favorable response (27). In a case of primary mediastinal choriocarcinoma, the patient initially achieved partial remission after two cycles of pembrolizumab but developed resistance. Despite attempting a combination treatment of pembrolizumab with chemotherapy, however, the treatment proved unsuccessful due to rapid disease progression (48). In contrast, a case involving primary neck choriocarcinoma achieved a complete response when treated with a combination of pembrolizumab and chemotherapy (49). The effectiveness of ICIs in non-gestational choriocarcinoma showed varying outcomes and requires additional research.

Regarding lung cancer with choriocarcinoma features, its rarity has led to a lack of clinical trial data on the efficacy of ICIs. Only a few case reports have utilized ICIs in this rare subtype of lung cancer. Buza et al. reported a case in which the patient initiated first line treatment with carboplatin and paclitaxel for 6 cycles. PD-L1 immunostaining showed a 30% staining of tumor cells. However, during the follow-up period, the tumor experienced progression, leading to the administration of pembrolizumab. Unfortunately, the disease continued to progress and the patient passed away (11). Another case report explored nivolumab as a second line treatment following the initial administration of pemetrexed/cisplatin/bevacizumab. PD-L1 immunostaining exhibited positivity in more than 50% of cells. The patient achieved a partial response after completing the first 4 cycles of immunotherapy, but CT indicated disease progression after 1 year of treatment (50).

The integration of immunotherapy with chemotherapy has emerged as the primary treatment approach for NSCLC. However, the potential application of this combined treatment in lung cancer with choriocarcinoma features or even as a first-line option remains uncertain. A case study implementing the CheckMate 9LA regimen was conducted after a patient exhibited postoperative relapse. Following two months of treatment, the patient achieved a partial response and subsequently received nivolumab and ipilimumab as part of maintenance therapy. This positive response persisted throughout the 12-month follow-up period (51). To our knowledge, the patient in our study represents the second reported instance of receiving combined anti-PD-1/anti-CTLA-4 therapy and chemotherapy in lung cancer with choriocarcinoma features. After one cycle of EP and two cycles of CheckMate 9LA, the primary tumor exhibited a reduction in size. Subsequent maintenance treatment involving eight cycles of nivolumab and ipilimumab, followed by nivolumab monotherapy, a continuous decrease in tumor size was observed, while other sites remained stable. Her relatively small disease burden also made it possible for us to offer LCT, which may also have contributed to her good disease control. These encouraging results suggest that the combination of chemoimmunotherapy with local radiation therapy may provide a promising therapeutic option for patients with lung cancer with choriocarcinoma features (**Figure 2C**).

## Conclusion

Lung cancer with choriocarcinoma features is a rare and aggressive malignancy. Determining the primary origin by traditional pathology assessment based on morphology and IHC is challenging. As a result, delayed diagnosis is common in patients with this rare subtype of lung cancer. In the era of precision medicine, the utilization of molecular profiling has proven highly informative and has significant diagnostic implications, particularly for cancer of rare histologies. The molecular information could not only facilitate identifying the origin of malignancy but may also guide decision making for treatment approaches on or off trials. For lung cancer with choriocarcinoma features, despite the lack of established treatment options, our case and others demonstrate the potential of chemoimmunotherapy in treating this subtype of lung cancer. In addition, local consolidation therapy could be considered for patients with small disease burden and good performance status.

## Data availability statement

The data presented in the study are deposited in the GEO repository, accession number GSE261551.

## Ethics statement

The studies involving humans were approved by The University of Texas MD Anderson Cancer Center. The studies were conducted in accordance with the local legislation and institutional requirements. The participants provided their written informed consent to participate in this study. Written informed consent was obtained from the individual(s) for the publication of any potentially identifiable images or data included in this article.

## Author contributions

HL: Data curation, Formal analysis, Writing – original draft, Writing – review & editing. XH: Formal analysis, Writing – review & editing. MN: Writing – review & editing. GF: Writing – review & editing. JS: Writing – review & editing. JG: Writing – review & editing. JW: Writing – review & editing. XL: Writing – review & editing. AV: Writing – review & editing. JL: Writing – review & editing. DG: Writing – review & editing. JH: Writing – review & editing. AF: Writing – review & editing. JZ: Conceptualization, Funding acquisition, Supervision, Writing – review & editing.
