# Supplementary material for: Case report: Molecular profiling facilitates the diagnosis of a challenging case of lung cancer with choriocarcinoma features
Source: Front Oncol. 2024 Mar 25;14:1324057. doi: 10.3389/fonc.2024.1324057 (PMC10999639; doi:10.3389/fonc.2024.1324057)
Supplement: Supplementary file 1 [file Table_1.docx]

Supplementary Material

Case Report: Molecular Profiling Facilitates the Diagnosis of a Challenging Case of Lung Cancer with Choriocarcinoma Features

Hui Li^1,2^, Xin Hu^3^, Matthew S Ning^4^, Gregory N Fuller^5^, John M Stewart^5^, Jared C Gilliam^6^, Jia Wu^1,2^, Xiuning Le^1^, Ara A Vaporciyan^7^, J. Jack Lee^8^, Don L Gibbons^1,9^, John V Heymach^1^, Andrew Futreal^3^, Jianjun Zhang^1,3*^

*** Correspondence:** Corresponding Author: [JZhang20@mdanderson.org](mailto:JZhang20@mdanderson.org)

**Supplementary Table 1.** Summary of reported cases with lung cancer with choriocarcinoma features in recent 5 years.

| First Author | Age | Sex | Smoking History | Symptom | Immunohistochemistry | Genetic Testing | Treatment | Outcome |
| --- | --- | --- | --- | --- | --- | --- | --- | --- |
| Buza N, et al(Case 2)(11) | 41 | Female | N/A | Irregular bleeding | CK AE1/AE3(+), β-hCG(+), GATA3(+), focally EMA(+), p40(+), PD-L1 30% | SMARCA4, ARID1A, NF2, ATR, CDK12, and POLE mutations | Chemotherapy, gamma knife to brain, pembrolizumab, right parietal craniotomy | Died 15 months after presentation |
| Chiang K, et al(52) | 47 | Female | Never | Hemoptysis | β-hCG(+), p40(+), MNF-116(+), K903(+), CK-7(+), PLAP(+) | N/A | Surgery, chemotherapy | Follow-up for 1 year without recurrence |
| Dlewati M, et al(53) | 54 | Female | N/A | Dyspnea, cough, intermittent hemoptysis | GATA-3(+), focally CDX2(+), Sal 4(+), pan-CK (+), CK 8/18(+), β-HCG (+), rare Glypican 1(+) | N/A | Neoadjuvant chemotherapy, surgery, adjuvant radiation therapy | Follow-up for 9 months with recurrence and metastasis |
| Iso H, et al(51) | 72 | Male | Heavy smoker | No symptoms | PD-L1 0% | No mutations | Surgery, combined chemotherapy and immunotherapy, nivolumab and ipilimumab maintenance | Follow-up for 12 months with partial response |
| Iwasaki K, et al(54) | 69 | Male | Light smoker | Left shoulder pain at first, then dyspnea and chest pain | β-hCG(+) | N/A | N/A | Died after 18 days of admission |
| Johnson A, et al (8) | 37 | Female | Never | Recurrent syncopal episodes | AE1/AE3(+), β-hCG(+) | N/A | Surgery, chemotherapy | Follow-up for 9 years without recurrence |
| Kim J, et al(33) | 44 | Female | N/A | Chest wall pain, a febrile sensation | β-hCG(+), CK-7(+), PAX-8(+), focally WT-1(+) | N/A | Chemotherapy | Follow-up for 2 years without recurrence |
| Ma Y, et al(28) | 71 | Male | Heavy smoker | Cough and hemoptysis | β-hCG(+), CK8(+), CK19(+) | STK11 c.291‑1G>C, TP53 D281E, SMARCA4 E1542*, PCNT E1491D, INPP4B N228K, ALK S639R, DNMT3A Y395C, INPP4B T671S | Surgery | Recurrence occurred 3 months after surgery and died |
| Matsukuma S, et al (Case 1)(55) | 53 | Male | N/A | Cough and chest pain | p63(+), p40(+) | N/A | Neoadjuvant chemoradiotherapy, surgery, chemotherapy | Died 15 months after presentation |
| Nguyen H,et al(15) | 42 | Male | Heavy smoker | Chest pain, weight loss | CK7(+), CKAE1/AE3(+), p40(+), SALL4(+), β-hCG (+) | N/A | Surgery, chemotherapy | Follow-up for 6 months without recurrence |
| Ochi M, et al (50) | 60 | Male | N/A | Headache, ataxia and dizziness | Only brain lesion β-hCG(+), PD-L1 > 50% | EGFR (-), ALK (-), ROS1(-) | Craniotomy, chemotherapy, nivolumab, surgery | N/A |
| Onishi L, et al(18) | 40 | Female | Heavy smoker | No symptoms | β-hCG(+), Glypican-3(+),p40(+) | MSI not detected, low TMB, EGFR V774M | Surgery | N/A |
| Shigematsu Y, et al(17) | 78 | Female | Never | Wet cough, exertional dyspnea | SALL4(+), β-hCG(+) | EGFR L858R, TP53 C275G | N/A | Died 11 days after presentation |
| Sousa M, et al(19) | 56 | Female | Heavy smoker | Dyspnea, dry cough, anorexia, and weight loss | CKAE1/AE3(+), vimentin(+), β-HCG(+), focally CK7(+),Ki-67 50%, PD-L1 10% | N/A | N/A | N/A |
| Stockton L, et al (56) | 39 | Female | Heavy smoker | Shortness of breath and pleuritic chest pain | CKAE1/AE3(+), Cytokeratin MNF116(+), CK7(+), CD10(+), P63(+), focal β-hCG (+) | N/A | Chemotherapy | Died after 11 day of admission |
| Wu P(57) | 37 | Female | Light smoker | Cough, dyspnea, chest pain, and hemoptysis with weight loss | CKAE1/AE3(+), β-hCG (+), HSD3B1(+), SALL-4(+) | N/A | Surgery, chemotherapy, Surgery again | Follow-up for 3 years without recurrence |
| Zhang X, et al(26) | 65 | Male | N/A | No symptoms | β-hCG (+), CATA-3(+), SMARCA4(+), CK7(+), CK(+), P40(+), PD-L1 1%-50% | TP53 R273L, NRAS amplification, FGFR1 E792fs | Surgery | N/A |

**References**

52. Chiang KY, Yan SW, Wong CF, Yeung MC, Chan GS, Hsin MK, et al. A 47-Year-Old Woman With Hemoptysis and a Lung Cyst. *Chest* (2020) 158(4):e159-e62. doi: 10.1016/j.chest.2020.05.547

53. Dlewati MM, Gonzalez T, Razi SS, Hussain SF, Bennett J. Primary Pulmonary Choriocarcinoma Treated With Neoadjuvant Chemotherapy and Lobectomy: A Case Report. *Cureus* (2022) 14(2):e21931. doi: 10.7759/cureus.21931

54. Iwasaki K, Watanabe K, Kimura H, Yano S. Utility of beta-human chorionic gonadotropin in pleural effusions: Report of an autopsy case of a male patient with primary pulmonary choriocarcinoma. *Clin Case Rep* (2022) 10(12):e6663. doi: 10.1002/ccr3.6663

55. Matsukuma S, Obara K, Utsumi Y, Miyai K, Takeo H, Oshika Y, et al. Focal positivity of immunohistochemical markers for pulmonary squamous cell carcinoma in primary pulmonary choriocarcinoma: A histopathological study. *Oncol Lett* (2018) 16(6):7256-63. doi: 10.3892/ol.2018.9525

56. Stockton L, Green E, Kaur B, De Winton E. Non-Gestational Choriocarcinoma with Widespread Metastases Presenting with Type 1 Respiratory Failure in a 39-Year-Old Female: Case Report and Review of the Literature. *Case Rep Oncol* (2018) 11(1):151-8. doi: 10.1159/000486639

57. Wu PS. Primary choriocarcinoma of the lung: a case report and literature review. *Int J Clin Exp Pathol* (2020) 13(9):2352-5.
